# Supplementary material for: Heparin-based hydrogel scaffolding alters the transcriptomic profile and increases the chemoresistance of MDA-MB-231 triple-negative breast cancer cells
Source: Biomater Sci. 2020 Feb 13;8(10):2786–96. doi: 10.1039/c9bm01481k (PMC7497406; doi:10.1039/c9bm01481k)
Supplement: Supplementary file 2 [file BM-008-C9BM01481K-s002.zip › Supplementary File 4/EGFvControl/Pathways/my_analysis.Gsea.1545200981068/HALLMARK_MYC_TARGETS_V1.html]

Details for gene set HALLMARK\_MYC\_TARGETS\_V1[GSEA]

|  || Dataset | expr.class.cls#EGF\_versus\_CONTROL.class.cls#EGF\_versus\_CONTROL\_repos |
| Phenotype | class.cls#EGF\_versus\_CONTROL\_repos |
| Upregulated in class | EGF |
| GeneSet | HALLMARK\_MYC\_TARGETS\_V1 |
| Enrichment Score (ES) | 0.6308562 |
| Normalized Enrichment Score (NES) | 2.8932834 |
| Nominal p-value | 0.0 |
| FDR q-value | 0.0 |
| FWER p-Value | 0.0 |
Table: GSEA Results Summary

  

Fig 1: Enrichment plot: HALLMARK\_MYC\_TARGETS\_V1      
 Profile of the Running ES Score & Positions of GeneSet Members on the Rank Ordered List

  

| PROBE | DESCRIPTION (from dataset) | GENE SYMBOL | GENE\_TITLE | RANK IN GENE LIST | RANK METRIC SCORE | RUNNING ES | CORE ENRICHMENT || 1 | NOP16 | na |  |  | 164 | 2.177 | 0.0026 | Yes |
| 2 | CAD | na |  |  | 345 | 1.896 | 0.0029 | Yes |
| 3 | ODC1 | na |  |  | 415 | 1.835 | 0.0087 | Yes |
| 4 | KARS | na |  |  | 427 | 1.823 | 0.0175 | Yes |
| 5 | NOLC1 | na |  |  | 443 | 1.809 | 0.0261 | Yes |
| 6 | RRP9 | na |  |  | 522 | 1.757 | 0.0310 | Yes |
| 7 | CDC20 | na |  |  | 590 | 1.712 | 0.0363 | Yes |
| 8 | HSPD1 | na |  |  | 619 | 1.691 | 0.0435 | Yes |
| 9 | CCT2 | na |  |  | 637 | 1.677 | 0.0513 | Yes |
| 10 | CCT5 | na |  |  | 653 | 1.669 | 0.0591 | Yes |
| 11 | CCNA2 | na |  |  | 725 | 1.626 | 0.0637 | Yes |
| 12 | C1QBP | na |  |  | 737 | 1.618 | 0.0715 | Yes |
| 13 | SNRPB2 | na |  |  | 742 | 1.616 | 0.0796 | Yes |
| 14 | NME1 | na |  |  | 764 | 1.603 | 0.0868 | Yes |
| 15 | MRPL9 | na |  |  | 766 | 1.602 | 0.0950 | Yes |
| 16 | DDX21 | na |  |  | 775 | 1.599 | 0.1028 | Yes |
| 17 | CDC45 | na |  |  | 795 | 1.593 | 0.1100 | Yes |
| 18 | IFRD1 | na |  |  | 799 | 1.591 | 0.1180 | Yes |
| 19 | AIMP2 | na |  |  | 810 | 1.586 | 0.1257 | Yes |
| 20 | EIF1AX | na |  |  | 823 | 1.578 | 0.1332 | Yes |
| 21 | PSMD14 | na |  |  | 825 | 1.577 | 0.1412 | Yes |
| 22 | PA2G4 | na |  |  | 838 | 1.574 | 0.1487 | Yes |
| 23 | SRSF1 | na |  |  | 863 | 1.566 | 0.1555 | Yes |
| 24 | TCP1 | na |  |  | 866 | 1.565 | 0.1635 | Yes |
| 25 | RUVBL2 | na |  |  | 873 | 1.563 | 0.1712 | Yes |
| 26 | MCM2 | na |  |  | 906 | 1.550 | 0.1775 | Yes |
| 27 | CCT7 | na |  |  | 916 | 1.546 | 0.1850 | Yes |
| 28 | SRM | na |  |  | 940 | 1.535 | 0.1917 | Yes |
| 29 | ABCE1 | na |  |  | 1023 | 1.506 | 0.1951 | Yes |
| 30 | IARS | na |  |  | 1033 | 1.502 | 0.2024 | Yes |
| 31 | TYMS | na |  |  | 1066 | 1.490 | 0.2084 | Yes |
| 32 | PHB | na |  |  | 1075 | 1.488 | 0.2156 | Yes |
| 33 | MCM7 | na |  |  | 1099 | 1.479 | 0.2221 | Yes |
| 34 | RAN | na |  |  | 1116 | 1.473 | 0.2288 | Yes |
| 35 | NOP56 | na |  |  | 1153 | 1.459 | 0.2344 | Yes |
| 36 | RRM1 | na |  |  | 1173 | 1.450 | 0.2409 | Yes |
| 37 | POLE3 | na |  |  | 1179 | 1.447 | 0.2481 | Yes |
| 38 | EIF3B | na |  |  | 1184 | 1.446 | 0.2553 | Yes |
| 39 | EIF3D | na |  |  | 1221 | 1.435 | 0.2608 | Yes |
| 40 | USP1 | na |  |  | 1270 | 1.423 | 0.2656 | Yes |
| 41 | CUL1 | na |  |  | 1288 | 1.415 | 0.2720 | Yes |
| 42 | NCBP1 | na |  |  | 1302 | 1.411 | 0.2786 | Yes |
| 43 | EPRS | na |  |  | 1365 | 1.389 | 0.2825 | Yes |
| 44 | ERH | na |  |  | 1380 | 1.384 | 0.2889 | Yes |
| 45 | TFDP1 | na |  |  | 1387 | 1.381 | 0.2957 | Yes |
| 46 | RNPS1 | na |  |  | 1393 | 1.380 | 0.3025 | Yes |
| 47 | RFC4 | na |  |  | 1417 | 1.372 | 0.3084 | Yes |
| 48 | TRA2B | na |  |  | 1432 | 1.369 | 0.3147 | Yes |
| 49 | EIF3J | na |  |  | 1474 | 1.355 | 0.3195 | Yes |
| 50 | STARD7 | na |  |  | 1544 | 1.335 | 0.3228 | Yes |
| 51 | GOT2 | na |  |  | 1607 | 1.316 | 0.3263 | Yes |
| 52 | MCM4 | na |  |  | 1617 | 1.313 | 0.3326 | Yes |
| 53 | PWP1 | na |  |  | 1637 | 1.310 | 0.3383 | Yes |
| 54 | ETF1 | na |  |  | 1652 | 1.307 | 0.3443 | Yes |
| 55 | MCM6 | na |  |  | 1672 | 1.301 | 0.3500 | Yes |
| 56 | MAD2L1 | na |  |  | 1695 | 1.296 | 0.3555 | Yes |
| 57 | PCNA | na |  |  | 1712 | 1.291 | 0.3613 | Yes |
| 58 | VBP1 | na |  |  | 1757 | 1.278 | 0.3656 | Yes |
| 59 | SRSF2 | na |  |  | 1835 | 1.260 | 0.3681 | Yes |
| 60 | XPO1 | na |  |  | 1845 | 1.259 | 0.3741 | Yes |
| 61 | SLC25A3 | na |  |  | 1903 | 1.246 | 0.3775 | Yes |
| 62 | KPNA2 | na |  |  | 1925 | 1.241 | 0.3828 | Yes |
| 63 | GNL3 | na |  |  | 1932 | 1.239 | 0.3888 | Yes |
| 64 | HDDC2 | na |  |  | 1934 | 1.239 | 0.3952 | Yes |
| 65 | IMPDH2 | na |  |  | 1989 | 1.223 | 0.3986 | Yes |
| 66 | XRCC6 | na |  |  | 1990 | 1.222 | 0.4049 | Yes |
| 67 | CLNS1A | na |  |  | 2008 | 1.217 | 0.4103 | Yes |
| 68 | SNRPD1 | na |  |  | 2237 | 1.171 | 0.4043 | Yes |
| 69 | KPNB1 | na |  |  | 2253 | 1.166 | 0.4096 | Yes |
| 70 | CCT3 | na |  |  | 2276 | 1.160 | 0.4144 | Yes |
| 71 | RPS3 | na |  |  | 2280 | 1.160 | 0.4202 | Yes |
| 72 | CDK2 | na |  |  | 2311 | 1.153 | 0.4246 | Yes |
| 73 | EXOSC7 | na |  |  | 2318 | 1.152 | 0.4302 | Yes |
| 74 | BUB3 | na |  |  | 2328 | 1.149 | 0.4356 | Yes |
| 75 | SF3B3 | na |  |  | 2347 | 1.145 | 0.4406 | Yes |
| 76 | PSMD1 | na |  |  | 2355 | 1.144 | 0.4461 | Yes |
| 77 | DDX18 | na |  |  | 2362 | 1.142 | 0.4517 | Yes |
| 78 | PABPC4 | na |  |  | 2525 | 1.107 | 0.4488 | Yes |
| 79 | PSMC4 | na |  |  | 2550 | 1.101 | 0.4533 | Yes |
| 80 | DHX15 | na |  |  | 2571 | 1.099 | 0.4579 | Yes |
| 81 | U2AF1 | na |  |  | 2593 | 1.095 | 0.4624 | Yes |
| 82 | HDAC2 | na |  |  | 2599 | 1.094 | 0.4678 | Yes |
| 83 | RSL1D1 | na |  |  | 2629 | 1.089 | 0.4719 | Yes |
| 84 | HNRNPC | na |  |  | 2655 | 1.083 | 0.4761 | Yes |
| 85 | COPS5 | na |  |  | 2665 | 1.082 | 0.4812 | Yes |
| 86 | PSMC6 | na |  |  | 2673 | 1.081 | 0.4864 | Yes |
| 87 | PSMA4 | na |  |  | 2701 | 1.076 | 0.4905 | Yes |
| 88 | HPRT1 | na |  |  | 2703 | 1.076 | 0.4960 | Yes |
| 89 | PHB2 | na |  |  | 2720 | 1.073 | 0.5007 | Yes |
| 90 | G3BP1 | na |  |  | 2756 | 1.068 | 0.5044 | Yes |
| 91 | SNRPA1 | na |  |  | 2766 | 1.066 | 0.5094 | Yes |
| 92 | ORC2 | na |  |  | 2772 | 1.065 | 0.5146 | Yes |
| 93 | EIF2S1 | na |  |  | 2819 | 1.058 | 0.5176 | Yes |
| 94 | EEF1B2 | na |  |  | 2842 | 1.054 | 0.5219 | Yes |
| 95 | PSMA7 | na |  |  | 2854 | 1.052 | 0.5268 | Yes |
| 96 | SNRPD2 | na |  |  | 2874 | 1.047 | 0.5312 | Yes |
| 97 | DEK | na |  |  | 2886 | 1.046 | 0.5360 | Yes |
| 98 | RANBP1 | na |  |  | 2912 | 1.042 | 0.5400 | Yes |
| 99 | CNBP | na |  |  | 2934 | 1.036 | 0.5443 | Yes |
| 100 | PTGES3 | na |  |  | 2955 | 1.034 | 0.5485 | Yes |
| 101 | RAD23B | na |  |  | 2977 | 1.030 | 0.5527 | Yes |
| 102 | XPOT | na |  |  | 2983 | 1.029 | 0.5578 | Yes |
| 103 | CCT4 | na |  |  | 3000 | 1.028 | 0.5622 | Yes |
| 104 | EIF4E | na |  |  | 3033 | 1.021 | 0.5658 | Yes |
| 105 | RPS2 | na |  |  | 3084 | 1.010 | 0.5684 | Yes |
| 106 | PPM1G | na |  |  | 3140 | 0.999 | 0.5706 | Yes |
| 107 | MCM5 | na |  |  | 3202 | 0.986 | 0.5725 | Yes |
| 108 | AP3S1 | na |  |  | 3272 | 0.974 | 0.5739 | Yes |
| 109 | VDAC3 | na |  |  | 3332 | 0.965 | 0.5757 | Yes |
| 110 | PRPS2 | na |  |  | 3412 | 0.951 | 0.5765 | Yes |
| 111 | TRIM28 | na |  |  | 3413 | 0.951 | 0.5814 | Yes |
| 112 | RPL6 | na |  |  | 3444 | 0.946 | 0.5847 | Yes |
| 113 | RPS5 | na |  |  | 3453 | 0.945 | 0.5891 | Yes |
| 114 | SYNCRIP | na |  |  | 3486 | 0.939 | 0.5923 | Yes |
| 115 | GLO1 | na |  |  | 3519 | 0.934 | 0.5954 | Yes |
| 116 | GSPT1 | na |  |  | 3557 | 0.928 | 0.5982 | Yes |
| 117 | ACP1 | na |  |  | 3596 | 0.921 | 0.6010 | Yes |
| 118 | PRDX3 | na |  |  | 3704 | 0.903 | 0.6000 | Yes |
| 119 | DUT | na |  |  | 3726 | 0.900 | 0.6035 | Yes |
| 120 | VDAC1 | na |  |  | 3760 | 0.892 | 0.6064 | Yes |
| 121 | EIF4H | na |  |  | 3786 | 0.886 | 0.6096 | Yes |
| 122 | MYC | na |  |  | 3907 | 0.866 | 0.6078 | Yes |
| 123 | POLD2 | na |  |  | 3987 | 0.852 | 0.6080 | Yes |
| 124 | H2AFZ | na |  |  | 4017 | 0.848 | 0.6109 | Yes |
| 125 | RPL18 | na |  |  | 4032 | 0.846 | 0.6145 | Yes |
| 126 | TUFM | na |  |  | 4052 | 0.843 | 0.6178 | Yes |
| 127 | CSTF2 | na |  |  | 4186 | 0.824 | 0.6151 | Yes |
| 128 | SNRPG | na |  |  | 4213 | 0.820 | 0.6179 | Yes |
| 129 | CBX3 | na |  |  | 4321 | 0.805 | 0.6165 | Yes |
| 130 | PSMB3 | na |  |  | 4433 | 0.788 | 0.6147 | Yes |
| 131 | SET | na |  |  | 4444 | 0.786 | 0.6182 | Yes |
| 132 | TARDBP | na |  |  | 4465 | 0.783 | 0.6212 | Yes |
| 133 | LSM2 | na |  |  | 4482 | 0.780 | 0.6244 | Yes |
| 134 | SRSF7 | na |  |  | 4523 | 0.774 | 0.6262 | Yes |
| 135 | FBL | na |  |  | 4582 | 0.766 | 0.6271 | Yes |
| 136 | ILF2 | na |  |  | 4735 | 0.741 | 0.6230 | Yes |
| 137 | HNRNPR | na |  |  | 4754 | 0.738 | 0.6258 | Yes |
| 138 | CDK4 | na |  |  | 4801 | 0.730 | 0.6272 | Yes |
| 139 | CYC1 | na |  |  | 4803 | 0.729 | 0.6309 | Yes |
| 140 | SRPK1 | na |  |  | 4989 | 0.709 | 0.6248 | No |
| 141 | SSB | na |  |  | 5162 | 0.683 | 0.6192 | No |
| 142 | SSBP1 | na |  |  | 5192 | 0.681 | 0.6212 | No |
| 143 | SERBP1 | na |  |  | 5255 | 0.671 | 0.6214 | No |
| 144 | HNRNPU | na |  |  | 5265 | 0.670 | 0.6244 | No |
| 145 | SRSF3 | na |  |  | 5273 | 0.669 | 0.6275 | No |
| 146 | PSMA1 | na |  |  | 5362 | 0.656 | 0.6262 | No |
| 147 | RPS6 | na |  |  | 5417 | 0.648 | 0.6267 | No |
| 148 | UBA2 | na |  |  | 5497 | 0.637 | 0.6259 | No |
| 149 | NAP1L1 | na |  |  | 5643 | 0.619 | 0.6214 | No |
| 150 | PSMA2 | na |  |  | 5669 | 0.614 | 0.6233 | No |
| 151 | RPL14 | na |  |  | 5716 | 0.607 | 0.6240 | No |
| 152 | HSPE1 | na |  |  | 5965 | 0.574 | 0.6139 | No |
| 153 | SF3A1 | na |  |  | 6095 | 0.557 | 0.6100 | No |
| 154 | EIF4A1 | na |  |  | 6111 | 0.553 | 0.6120 | No |
| 155 | NCBP2 | na |  |  | 6120 | 0.552 | 0.6144 | No |
| 156 | PRDX4 | na |  |  | 6214 | 0.538 | 0.6123 | No |
| 157 | UBE2L3 | na |  |  | 6434 | 0.510 | 0.6034 | No |
| 158 | EIF2S2 | na |  |  | 6459 | 0.507 | 0.6048 | No |
| 159 | YWHAE | na |  |  | 6474 | 0.506 | 0.6067 | No |
| 160 | PSMD7 | na |  |  | 7032 | 0.443 | 0.5796 | No |
| 161 | TXNL4A | na |  |  | 7384 | 0.398 | 0.5632 | No |
| 162 | FAM120A | na |  |  | 7511 | 0.385 | 0.5586 | No |
| 163 | HNRNPD | na |  |  | 7535 | 0.381 | 0.5593 | No |
| 164 | HNRNPA1 | na |  |  | 7833 | 0.346 | 0.5455 | No |
| 165 | PSMB2 | na |  |  | 8011 | 0.324 | 0.5378 | No |
| 166 | MRPS18B | na |  |  | 8444 | 0.280 | 0.5166 | No |
| 167 | NDUFAB1 | na |  |  | 8463 | 0.277 | 0.5170 | No |
| 168 | SNRPA | na |  |  | 8730 | 0.242 | 0.5043 | No |
| 169 | PSMD3 | na |  |  | 9027 | 0.212 | 0.4898 | No |
| 170 | HNRNPA2B1 | na |  |  | 9586 | 0.147 | 0.4612 | No |
| 171 | RPL34 | na |  |  | 9791 | 0.126 | 0.4511 | No |
| 172 | HNRNPA3 | na |  |  | 9855 | 0.121 | 0.4484 | No |
| 173 | RPS10 | na |  |  | 9930 | 0.111 | 0.4451 | No |
| 174 | SNRPD3 | na |  |  | 10208 | 0.080 | 0.4310 | No |
| 175 | SMARCC1 | na |  |  | 10226 | 0.078 | 0.4305 | No |
| 176 | YWHAQ | na |  |  | 11309 | -0.041 | 0.3738 | No |
| 177 | PRPF31 | na |  |  | 11818 | -0.102 | 0.3476 | No |
| 178 | APEX1 | na |  |  | 11959 | -0.120 | 0.3408 | No |
| 179 | PCBP1 | na |  |  | 12190 | -0.142 | 0.3295 | No |
| 180 | COX5A | na |  |  | 13092 | -0.256 | 0.2834 | No |
| 181 | PSMD8 | na |  |  | 13461 | -0.309 | 0.2656 | No |
| 182 | UBE2E1 | na |  |  | 13628 | -0.336 | 0.2586 | No |
| 183 | HDGF | na |  |  | 14471 | -0.440 | 0.2166 | No |
| 184 | NHP2 | na |  |  | 15612 | -0.608 | 0.1597 | No |
| 185 | MRPL23 | na |  |  | 15933 | -0.673 | 0.1464 | No |
| 186 | RPL22 | na |  |  | 16805 | -0.888 | 0.1051 | No |
| 187 | PSMA6 | na |  |  | 16949 | -0.935 | 0.1024 | No |
| 188 | LSM7 | na |  |  | 17011 | -0.954 | 0.1041 | No |
| 189 | PGK1 | na |  |  | 18818 | -2.107 | 0.0200 | No |
Table: GSEA details [plain text format]

  

Fig 2: HALLMARK\_MYC\_TARGETS\_V1      
 Blue-Pink O' Gram in the Space of the Analyzed GeneSet

  

Fig 3: HALLMARK\_MYC\_TARGETS\_V1: Random ES distribution      
 Gene set null distribution of ES for **HALLMARK\_MYC\_TARGETS\_V1**
